# Design of a sustainable system for wastewater treatment and generation of biofuels based on the biomass of the aquatic plant *Eichhornia Crassipes*

**DOI:** 10.1038/s41598-024-61239-4

**Published:** 2024-05-14

**Authors:** Uriel Fernando Carreño Sayago, Melva Inés Gómez-Caicedo, Álvaro Luis Mercado Suárez

**Affiliations:** 1 Fundación Universitaria los Libertadores, Bogotá, Colombia; 2 Faculty of EconomicAdministrative and Accounting Sciences, Fundación Universitaria los Libertadores, Bogotá, Colombia

**Keywords:** E. *crassipes*, Phytoremediation, Bioethanol, Biohydrogen, Ecology, Environmental sciences, Engineering

## Abstract

Colombia’s continuous contamination of water resources and the low alternatives to produce biofuels have affected the fulfillment of the objectives of sustainable development, deteriorating the environment and affecting the economic productivity of this country. Due to this reality, projects on environmental and economic sustainability, phytoremediation, and the production of biofuels such as ethanol and hydrogen were combined. The objective of this article was to design and develop a sustainable system for wastewater treatment and the generation of biofuels based on the biomass of the aquatic plant *Eichhornia crassipes*. A system that simulates an artificial wetland with live *E. crassipes* plants was designed and developed, removing organic matter contaminants; subsequently, and continuing the sustainability project, bioreactors were designed, adapted, and started up to produce bioethanol and biohydrogen with the hydrolyzed biomass used in the phytoremediation process, generating around 12 g/L of bioethanol and around 81 ml H_2_/g. The proposed research strategy suggests combining two sustainable methods, bioremediation and biofuel production, to preserve the natural beauty of water systems and their surroundings.

## Introduction

Today, the world is facing a hydric crisis due to the lack of potable fresh water. Such hydric scarcity is a consequence of the rapid growth of cities and the large amount of domestic wastewater discharged into rivers and running water systems; the environmental, social, and health impacts of these pollutants are often incalculable^1^. Domestic and industrial wastewater treatment is usually very expensive, and because of it, companies irresponsibly choose not to treat their effluents, increasingly contaminating hydric sources. For this reason, budget and efficient technologies are required for the treatment of different types of water^2^. Environmental sustainability strategies that combine projects that generate impact and are disruptive must be sought in order to be easily implemented and thus favor ecosystems, preserving their natural beauty and contributing to increasing sustainable tourism^3,4^.

An example of this is the sustainable manipulation of the aquatic plant *E. crassipes,* which has been the source of numerous investigations around the world, such as the phytoremediation of polluted water and energy production^5–7^.

*E. crassipes* plant’s biomass can effectively treat wastewater by oxygenating the water and degrading organic matter. In recent years, sustainable handling of this species has shown practical solutions for designing water treatment systems that take advantage of its growth conditions. It results in a high degree of treatment, reducing DBO, nitrogen, and phosphorus levels^8^. Also, this same biomass can be transformed into biofuels, such as bioethanol and biohydrogen^9–12^. These biotechnologies could help the country in two important areas: water treatment and energy generation. Ethanol production from lignocellulosic material has become an interesting alternative for revaluing waste and opening new markets^13–15^. *E. crassipes* biomass has also been successfully used for biohydrogen production, with different bioreactors used depending on the metabolic bioprocess and microorganism type, including dark fermentation^16–19^. Anaerobic fermentation is a well-known method for producing biohydrogen^20^.

The aim of this article is to merge environmental sustainability projects by enhancing the implementation of life cycle analysis (LCA)^21–23^ for integrating circular economy practices in self-sustainable farms through phytoremediation and biofuel production. The plant material of *E. crassipes* was used to treat domestic wastewater and then utilized as a source for producing ethanol and hydrogen in fermentation bioreactors. The *E. crassipes* plant underwent a physicochemical characterization process to determine the present percentages of cellulose, hemicellulose, and lignin.

## Methods and materials

### Using *crassipes*

The leaves and roots were separated from the rest of the plant, washed with tap water, followed by distilled water, where a characteristic population of about 40 already dead plants was collected. The collection point is the municipality of La Palma, Cundinamarca, Colombia, located at the coordinates: 5.3605555555556, -74.389722222222.

The experimental research on plants, including the collection of plant material, complied with the relevant institutional, national, and international guidelines and legislation, as stipulated in Decree Law 2376 of 201323^24^, for experimental projects in the environment.

### The taxonomic level is *(Eichhornia crassipes)*

The collection point is the municipality of Mosquera, in the outskirts of Bogotá DC, located at the coordinates 4.682995, − 74.256673; this activity was carried out on June 15, 2022, by the researcher Uriel Fernando Carreño Sayago. The plant samples were also identified by the researcher at the Faculty of Engineering of Bogotá, D.C. The final biomass of the plants was used as input for composting processes at the Libertadores University, with the code LIB 021212.

### Characterization of *Eichhornia crassipes*

The physicochemical characterization was carried out to identify the properties of the collected macrophytes, determining the structural carbohydrates and the lignin content. In addition, the quantification of the biomass matrix used was carried out considering the following parameters: (a) % hemicellulose, (b) % cellulose, (c) % lignin, and (d) ashes^25–27^.

An extract of benzene and ethanol with a 2:1 volume ratio was used to extract cellulose. Nitric acid and ethanol were used in a 1:4 ratio to extract hemicellulose. For the extraction of lignin, 12% hydrochloric acid was used, and for the determination of ashes, 72% hydrogen sulfide was used.

### Phase 1. Assembly of the artificial wetland with the *E. crassipes*

**Figure 1:**
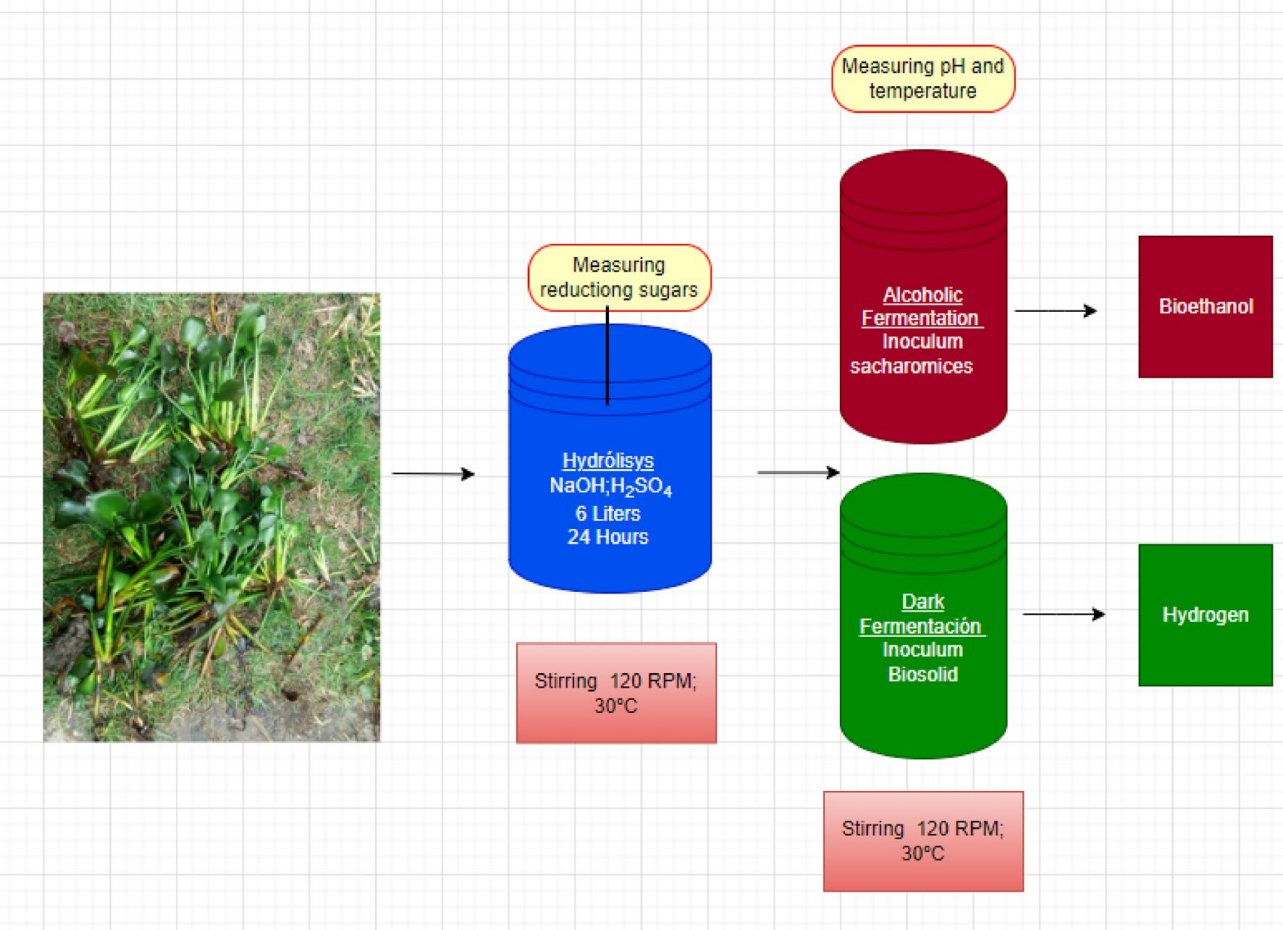
Experimental setup.

The dimensions of the experimental model of phytoremediation are 100 cm long and 80 cm tall. This design is on a pilot scale and has 2.5 kg of *E. crassipes* (approximately 25 plants). Figure 1 shows the treatment system. The experiment was carried out in triplicate (showing the average in the results), evaluating DBO, total nitrogen, Kendal nitrogen and phosphorus for 10 days, taking samples before and after the treatment. About 150 L of domestic wastewater were treated. Then, a plastic mesh that floats was designed and built, as well as treatment compartments where the *E. crassipes* plants are located.

Once the composite sample of the original residual water was obtained, it was characterized using ex-situ physicochemical parameters such as biochemical oxygen demand (DBO5), phosphorus, ammoniacal nitrogen, and total nitrogen. The analysis was carried out in a credited laboratory in Bogotá, following the protocols presented in the standardized methods and established in the most recent editions of the standard methods for the analysis of water and wastewater of the American Public Health Association (APHA) and the American water services association (AWWA). The cost of this system is around two hundred dollars.

### Phase 2. Production of ethanol and hydrogen

The design of the bioethanol and biohydrogen generation process consists of three bioreactors: a bioreactor to make the hydrolyzate, a bioreactor for fermentation generating bioethanol and dark fermentation. The following figure represents the joint process of hydrolysis and dark fermentation.

The design of the bioethanol and biohydrogen generation process has three bioreactors: a bioreactor to produce the hydrolyzate, a bioreactor for fermentation generating bioethanol, and the last bioreactor for dark fermentation. The following figure represents the joint process of hydrolysis and fermentations.

### Hydrolysis of *Eichhornia Crassipes*

The hydrolyzate bioreactor is made of glass, with a capacity of 6 L; it has a hose for the evolution of gases, pH, and temperature sampling, and it was placed in a heater with magnetic stirring at 120 rpm at a temperature of 30° C. In this bioreactor, 2.5 kg of dried and crushed *Eichhornia crassipes* was taken and mixed with distilled water^27,28^. The cost of this system is around one hundred dollars.

### Hydrolysis alkali

The samples of the *E. crassipes* were set to react in 1% (w/v) caustic soda (NaOH) at a temperature of 30° C for 12 h; then, the samples were washed with tap water until reaching the pH value of the water^27,28^.

### Hydrolysis acid

3% (v/v) sulfuric acid (H_2_SO_4_) was added at a temperature of 60° C, for 12 h. The samples were washed with tap water until reaching the pH value of water.

The content of reducing sugars was determined by the Dinitro Salicylic Acid (DNS) method, which indirectly quantifies the consumption of substrate. 6 L of *Eichhornia crassipes* hydrolyzate solution were obtained for the continuation of biofuel production^27,28^.

### Fermentation bioreactor

Glassware 2.5 L. The hydrolyzed plant material of *E.* crassipes (1 kg) was washed and taken to the alcoholic fermentation bioreactor, where 150 g of *Saccharomyces cerevisiae* was added; the pH should be around 6.0. The bioreactors were hermetically sealed with rubber septa and aluminum stoppers. During the hydrolyze fermentation, the tests of the ethanol percentages are carried out^28,29^. The experiment was carried out in duplicate (showing the average in the results). The cost of this system is around one hundred dollars.

### Production of biohydrogen

The dark fermentation bioreactor is made of glass, with a capacity of 4 L. It has a lid for gas release, pH, and temperature sampling, and it was placed in a heater with magnetic stirring at 120 RPM at a temperature of 30° C. The bioreactor was hermetically sealed with rubber septa and aluminum stoppers. Bird manure was used as raw material to carry out the hydrogen production process. After that, they were put to a temperature of 100° C in an oven to deactivate microorganisms that do not benefit the production process of this biofuel.

500 g of the hydrolyzate from the *E. crassipes* biomass were taken to the bioreactor, where it was mixed with distilled water, and 500 g of the inoculum (bird manure) was added; the initial pH was adjusted to 5.5. The bottle holes were purged with nitrogen for 5 min to ensure the anaerobic condition. At each time interval, the biogas volume was measured by the plunger displacement method. Hydrogen gas was determined by gas chromatography using a TCD detector on a GC-Agilent 7890 chromatograph. The optimum temperature for hydrogen production is 30° C. The experiment was carried out in duplicate (showing the average in the results). The cost of this system is around two hundred dollars.

The results of the different tests were determined with the Gompertz equation (Eq. (1))

1 $$H = Hmax \times exp + \left( {\left( { - \exp \left( {\frac{Rmax \times exp}{{Hmax}}} \right)} \right)} \right.\left( {\alpha - t} \right) + 1),$$

where, α latency time, R_m_ Maximum rate of H_2_ production, H_max_ Maximum production potential.

## Result

### Result of characterizations chemistry

The *E. crassipes* collected in the wet bodies had a hemicellulose content of 33% and 30% cellulose; lignin was lower, with 9%, and ash content was high, with 23% due to the contamination inherent to the plant. In Table 1 is the composition of the biomass of *E. crassipes.*

**Table 1:** Composition of the biomass of *E. crassipes.*

Lignine (%)	Cellulose (%)	Hemicellulose (%)	Other* (%)	Reference
9	23	43	23	Present
1.1	17.3	24.7		^25^
4.1	19.7	27.1		^26^
3.5	18.2	48.7	13.3	^27^
1.1	17.3	24.7		^28^
11	31	27	10	^29^
11	27	27	10	^30^
12	36	42		^31^

In different studies carried out where the cellulose of *E. crassipes* has been physicochemically characterized, the high presence of cellulose and hemicellulose in its chemical composition has been evidenced; such is the case of Refs.^25–29^ who averaged 18% in hemicellulose and 25% in cellulose. The presence of these two polysaccharides favors biofuel production^30^. The presence of lignin also makes the biomass of this plant a process extra of hydrolysis before the bioethanol and biohydrogen production process^31^.

### Analysis of phytoremediation

**Figure 2:**
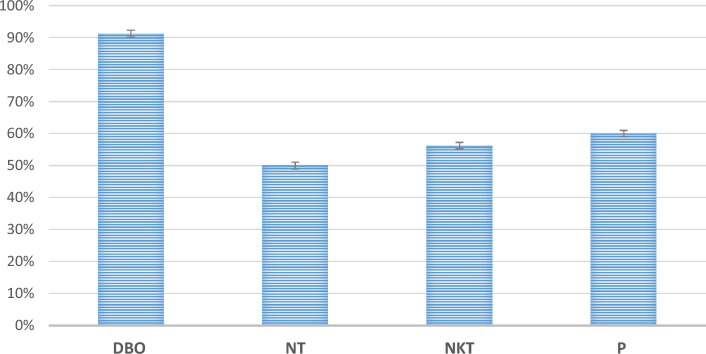
Percentages of removals in the Wetland with E. *crassipes*.

In the system of phytoremediation, the waste water had a very strong odor; however, through the phytoremediation process, there was a gradual reduction of it. Figure 2 shows the percentages of removals in the wetland with *E. crassipes.*

### Biochemical oxygen demand

The variation of the gross DBO of the influent and effluent samples of the systems can be observed in Fig. 2. The average removal efficiencies for DBO were 90%. The availability of oxygen in the wetland corresponds to the photosynthesis process carried out by the E. crassipes, being the amount of this plant essential to guarantee aerobiosis^22^. Aerobic conditions are necessary to reduce contamination by organic matter. The availability of oxygen is a design criterion for wetlands with E. crassipes, being essential for the biochemical removal of organic matter. 34 In a water treatment system from a tannery, were obtained removal efficiencies of 88% with E. crassipes plants in DBO^33,34^; they achieved a higher DBO removal efficiency (92.3%) in wetlands planted with *Phragmites australis* and *Canna indica*. Although the presence of chromium was minimal, it can still affect the efficiency of nutrient elimination. Heavy metals like chromium can inhibit root oxygenation and seriously impact the elimination of nitrogen and phosphorus^35–37^. To ensure effective treatment, it is important to separate domestic wastewater from industrial wastewater^38,39^.

### Nitrogen

The elimination of 50% of the nitrogen obeys to the fact that the plant incorporates nitrogen for its growth and subsequent reproduction^40^. The nitrogen present in domestic wastewater is organic and is transformed by hydrolysis into ammonia, followed by a chemical oxidation of the ammonia to nitrite and nitrate, the form in which it is assimilated by the plant^41^. The activity of certain anaerobic bacteria present in the roots of the plant leads to denitrification, which consists in the reduction of the nitrate ion to gaseous nitrogen, which is released to the atmosphere. The efficiency of Kjeldahl nitrogen is close to 55% in this type of wetland with *E. crassipes* because the plant adsorbs this nutrient mainly as ammonium and nitrate. A mixture of both forms is usually beneficial. These two forms of nitrogen differ in the way they are converted to amino acids in their metabolism in the plant. The ammonium is metabolized in the roots and requires more oxygen, while the nitrate metabolism takes place in the leaves due to the oxygenation of the water, and there is a mutual benefit between the treatment and the plant. Also, ammonium and nitrate uptake affect the root environment differently from another nutrient uptake^42,43^.

### Phosphorus

**Figure 3:**
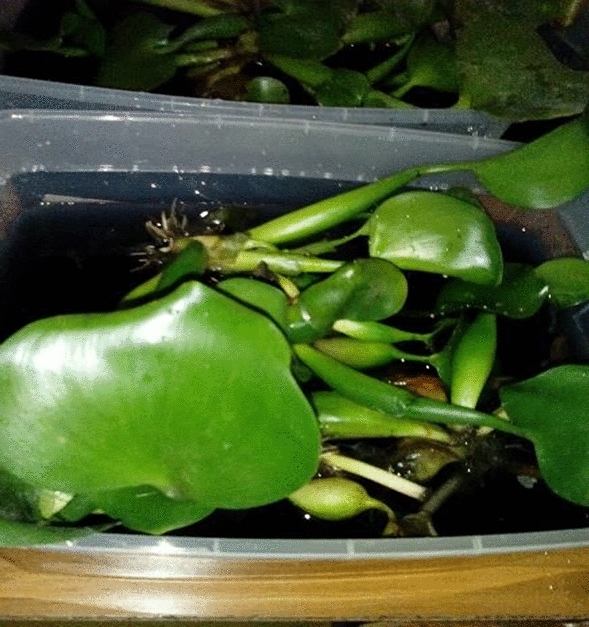
Biomass used in phytoremediation and subsequent biofuel processing.

The efficiencies report 60% because the removal process of this nutrient depends to a great extent on the bacteria present in the plant. Dissolved organic phosphorus, particulate organic phosphorus, and insoluble phosphorus are not available to plants unless they are transformed into soluble inorganic phosphorus^41,44^. In the *E. crassipes* wetland, these transformations can occur through the intervention of bacteria associated with the roots and in biofilms in the sediments. Once solubilized by these microorganisms, it can be assimilated by the plant for its growth and reproduction; being this process the treatment that the wetland has to reduce the phosphorus in the water^45,46^. The biomass used in the previous phytoremediation process was used in this biofuel production process (see Fig. 3).

### Hydrolysis results

There was a continuous production of sugars through acid hydrolyzation, alkaline hydrolyzation, and a combination between both processes. The best sugar production yield was the alkaline hydrolyzation, since it obtained a production of 140 g/L, and the acid hydrolyzation obtained a low production of around 60 g/L. The mixture between the two processes gave a result of 220 g/L. In the yield of sugar production, the alkaline hydrolyzation is the one that has the best performance with the *E. crassipes* plant^47,48^.

### Production of bioethanol

**Figure 4:**
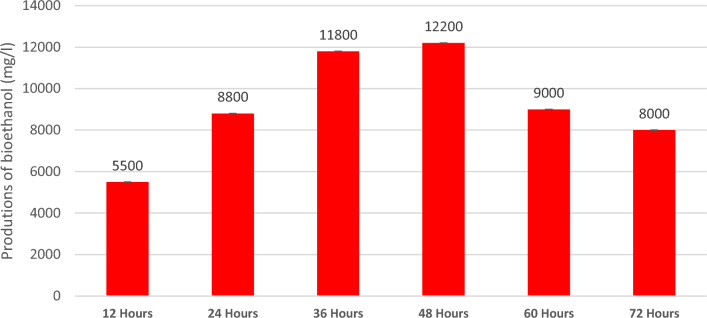
Production of bioethanol with the biomass of E. *crassipes.*

Figure 4 shows a higher ethanol production for the *E. crassipes* sample. When carrying out the mass balance, it was established that the production of ethanol from hydrolyzed biomass of *E. crassipes* is profitable, with an amount of 12,200 (mg/l) in 48 h, with a conversion of 90% of the sugars into ethanol, conversion results similar to Bioethanol production in Ref.^49^.

In the Table 2, show the resume of yield the biomass, where different investigations were taken in order to compare the results obtained.

**Table 2:** Process of productions bioetanol.

	Biomass	Yield (mg/l)
Present research	*Crassipes*	12,200
^52^	Cassava pulp	15,000
^53^	Watermelon	12,000
^54^	Potato peel wastes	22,000
^49^	*Crassipes*	15,000
^50^	*Pichia stipitis*	36,000
^51^	Lignocellulosic biomass	20,000
^55^	Pistia stratiotes	25,000
^56^	Banana waste	28,000
^57^	Macroalgae	18,000
^58^	Genetic modification of cereal plants	22,000
^59^	Carrot pulp	20,000
^60^	Cotton spinning	18,000
^61^	Pretratment Enzimatic (E. *crassipes*)	25,000
^62^	Treatment with TiO_2_ (E. *crassipes*)	26,000
^63^	Modification of soil bacterial	25,000

The biomass of the cellulose is a promising source of biofuel production; processes must be optimized to make better use of these resources. For example, Ref.^50^ used alkaline pretreated sugarcane bagasse using *Zymomonas mobilis* and *Pichia stipitis* in the fermentation, achieving a yield and ethanol productivity of 36,000 mg/l, (it/they). Also^51^ used lignocellulose biomass to generate bioethanol, with a yield of 20,000 mg/l using genetically modified yeasts. The cassava pulp yield was 15,000 mg/l of bioethanol^52^, peel has a yield of 12,000 mg/l^53^, and 22,000 mg/l were obtained from potato peel wastes^54^. But all these biomasses are not by-products, let alone having been used in other sustainable processes. The production of bioethanol with the *E. crassipes* plant after a phytoremediation process makes this process viable. The projects^55–63^ have interesting results in the productions of this biofuel, but with a more specialized process and increased cost, which makes production on a larger scale unfeasible. Although the production of bioethanol is not as high as in other investigations, the E. *crassipes* plant is a waste product and is also a biomass that was previously subjected to a phytoremediation process, which may have depleted the cellulose content of the plant.

### Hydrogen productions

**Figure 5:**
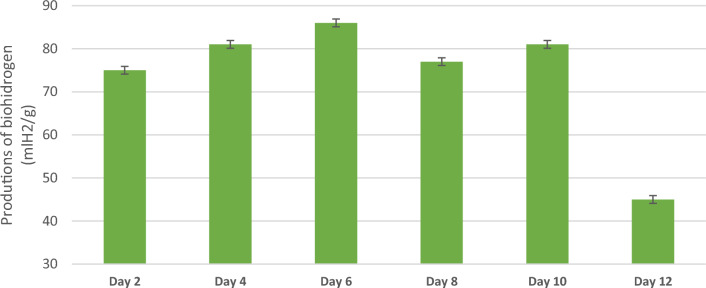
Production of biohydrogen with the biomass of E. *crassipes.*

The hydrogen gas yield production remained almost constant for 12 days, after which it decreased to half its value when the initial content of the inoculum was consumed, (from 80 to 40 ml H_2_/g glucose). Figure 5 shows the results of biohydrogen production for 10 continuous days of productivity.

The specific hydrogen production rate reached its maximum value (81.3 ml H_2_/g) on day 4. The production should be maintained consistently over the next few days, with a target of around 80.0 ml H_2_/g until day 10. From day 12 onwards, the production should be reduced to reach a balance of 40 ml H_2_/g. In the trials to produce biohydrogen from E. crassipes, around 73 ml H_2_/g was also produced^64^.

Biohydrogen production has been carried out using various substrates. For instance, in a study by^65^, biohydrogen was produced from the saccharification of alfalfa, resulting in a yield of 55 ml H_2_/g. Table 3 provides a summary of the biohydrogen production process.

**Table 3:** Process of productions of biohydrogen.

	Biomass	Yield H_2_/g
Present research	*Crassipes*	81.7
^64^	*Crassipes*	73
^65^	Alfalfa	55.6
^66^	Cane bagasse	59
^67^	*Alternanthera hiloxeroides*	100.1
^68^	cellulose	102.6
^69^	Cellulomonas biazotea	105.5
^70^	Optimizations microbial	106.7
^71^	Composite with *Crassipes*	100.2
^16^	Biomass modificated with nickel ferrite nanoparticles	104
^72^	Nanomaterials	105
^73^	Nanotechnology	118

In more specialized process, as in the case of Ref.^67–69^, the celluloses were modified genetically and hydrogen production was better, achieving biohydrogen production yields above 100 ml H_2_/g. It has been proven that the biomass of *E. crassipes* and other lignocellulolytic modified materials or with other components can also increase the production of biohydrogen^16,70–73^.

A residue remains in this process, which is a mixture of the *E. crassipes* plant and poultry manure. This material is sanitized and has potential as a biofertilizer due to its physicochemical characteristics^74–76^.

The anaerobic fermentation of organic matter produces an organic residue with excellent fertilizing properties. On average, the biofertilizer composition is 8.5% organic matter, 2.6% nitrogen, 1.5% phosphorus, 1.0% potassium, and has a pH of 7.5^77–79^.

### Life cycle analysis (LCA)

**Figure 6:**
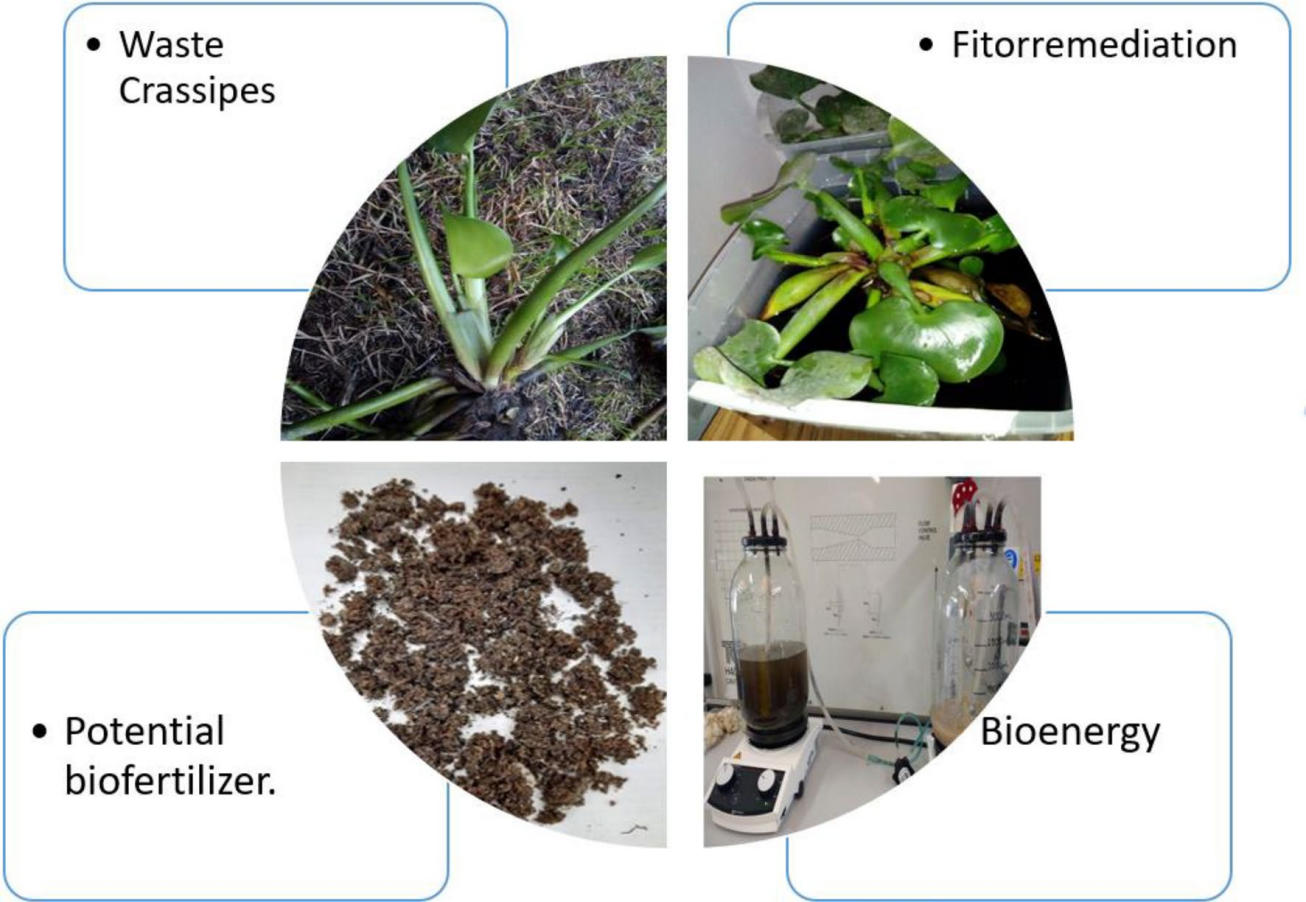
Life cycle analysis (LCA).

The biomass generation of the E. *crassipes* plant is considerable, with an estimated yield of approximately 30 tons per year in the city of Bogotá^32,38,80^. This makes it an ideal raw material for the development of phytoremediation and bioenergy generation systems, including bioethanol and biohydrogen. Figure 6 presents a summary of the Life Cycle Analysis (LCA) of the biomass of E. *crassipes.*

The generated product could be used as a potential fertilizer, thus furthering this research and generating technical and economic feasibility. It is imperative to implement this project on sustainable farms in our country, especially where the aquatic plants come from, La Palma Cundinamarca, Colombia.

## Conclusions

The research combined projects on environmental sustainability, phytoremediation, and biofuel generation. The *E. crassipes* plant material was used to treat domestic wastewater, which was then utilized as a source of ethanol and green hydrogen production in fermentation bioreactors.

The effectiveness of domestic wastewater treatment in removing organic matter represented by BOD was 90%. Total nitrogen removal was 50%; N_t_ nitrogen removal was 40%, and phosphorus removal was 60%. These results suggest that these nutrients were assimilated as a food source by *E. crassipes* plants. The constructed wetland is designed to meet treatment needs, and it can be used in various environments, such as farms, homes, and apartments, as an environmentally sustainable solution due to its effectiveness, ease of installation, and low cost.

As part of the sustainability project, the biomass used in the phytoremediation process underwent hydrolysis with H_2_SO_4_ and NaOH, producing 150 mg/L of available sugars. These sugars were then divided into two processes: alcoholic fermentation and dark fermentation.

A bioreactor was designed, adapted and launched for the production of bioethanol from the hydrolyzed biomass. The bioreactor produced approximately 12 g/L of bioethanol.

Furthermore, a bioreactor was designed and adapted for the production of biohydrogen from hydrolyzed biomass obtained from the same phytoremediation process. The bioreactor produced approximately 81 ml of H_2_/g. Additionally, organic waste from poultry manure was utilized in this process, and an organic fertilizer was also produced. Moreover, a domestic wastewater treatment system was developed using biomass that is typically discarded. This system produces two types of biofuels, bioethanol and biohydrogen, making it sustainable and profitable for large-scale implementation. It contributes to the improvement of Life Cycle Analysis (LCA) processes on a self-sustainable farm and is decisive in the concepts of circular economy. The development of these sustainable activities can recover and value water systems while also producing bioenergy with high efficiency; this establishes a synergy between bioremediation and the generation of biofuels.

## Data Availability

The datasets used and/or analyzed during the current study available from the corresponding author on reasonable request.
