# Author Correction: Design of a sustainable system for wastewater treatment and generation of biofuels based on the biomass of the aquatic plant *Eichhornia Crassipes*

**DOI:** 10.1038/s41598-024-63402-3

**Published:** 2024-06-04

**Authors:** Uriel Fernando Carreño Sayago, Melva Inés Gómez‑Caicedo, Álvaro Luis Mercado Suárez

**Affiliations:** 1 Fundación Universitaria los Libertadores, Bogotá, Colombia; 2 Faculty of EconomicAdministrative and Accounting Sciences, Fundación Universitaria los Libertadores, Bogotá, Colombia

Correction to: *Scientific Reports* 10.1038/s41598-024-61239-4, published online 14 May 2024

In the original version of this Article, the Funding section was incomplete. It now reads:

Funding

"The government of Cundinamarca financed this research within the framework of the project Development of a Sustainable Tourism Promotion Model from Technology 4.0, in the Municipalities of La Palma and Yacopí in the Department of Cundinamarca. The University Los Libertadores contributed to developing this article and other processes.”

The original Article has been corrected.

